# Mining Rare Associations between Biological Ontologies

**DOI:** 10.1371/journal.pone.0084475

**Published:** 2014-01-03

**Authors:** Fernando Benites, Svenja Simon, Elena Sapozhnikova

**Affiliations:** Department of Computer and Information Science, University of Konstanz, Konstanz, Germany; Saint Louis University, United States of America

## Abstract

The constantly increasing volume and complexity of available biological data requires new methods for their management and analysis. An important challenge is the integration of information from different sources in order to discover possible hidden relations between already known data. In this paper we introduce a data mining approach which relates biological ontologies by mining cross and intra-ontology pairwise generalized association rules. Its advantage is sensitivity to rare associations, for these are important for biologists. We propose a new class of interestingness measures designed for hierarchically organized rules. These measures allow one to select the most important rules and to take into account rare cases. They favor rules with an actual interestingness value that exceeds the expected value. The latter is calculated taking into account the parent rule. We demonstrate this approach by applying it to the analysis of data from Gene Ontology and GPCR databases. Our objective is to discover interesting relations between two different ontologies or parts of a single ontology. The association rules that are thus discovered can provide the user with new knowledge about underlying biological processes or help improve annotation consistency. The obtained results show that produced rules represent meaningful and quite reliable associations.

## Introduction

There has been an exponential increase in the volume and complexity of available biological data, which needs to be addressed with new methods of data management and analysis. Due to the fact that ontologies or hierarchies of concepts facilitate the exploration of information and its storage and understanding, they have been established as a standard in biology. Many biomedical ontologies have been developed for different domains. In the field of genomics the most famous example is the Gene Ontology (GO)(http://www.geneontology.org/). It provides a hierarchy of properties and functional categories for millions of genes and proteins, which are annotated as belonging to one or more categories (GO terms).

An important challenge of modern data analysis is caused by the variety of existing interrelated data sources, which all can be used to describe the same problem. Using different data sources is especially important in biological applications since a single data source can often reveal only a certain perspective of the underlying complex biological mechanism. Furthermore, many single-source-based approaches have been criticized for their low reliability [1]. In the last years, the bioinformatics community has encountered the need to integrate information in order to put the data in a useful context, extracting as much knowledge as possible [2]–[5].

In this regard, we are interested in discovering relationships between categories of different biological ontologies or different parts of a single ontology as in the case of three sub-ontologies of GO: Cellular Component (CC), Biological Process (BP) and Molecular Function (MF). While ontologies help scientists organize massive amounts of information, the problem of relating them is left open due to the lack of proper methods for knowledge integration. To solve it, data mining techniques such as association analysis may help explore dependencies between multiple ontologies that provide different insights into a certain problem. The subsequent combination of information can lead to the discovery of possibly unexpected knowledge.

Initially, association analysis was applied to the search for sets of elements that frequently co-occur in a transaction database and establishing relationships between them. A transaction is a set of items, for example, items purchased together. Co-occurring items build an Association Rule (AR) of the form 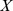 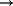 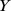, where 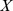 and 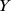 are sets of items (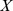 is called the antecedent and 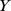 – the consequent). The support of a rule corresponds to the frequency of the co-occurrences of 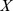 and  in the data; the support of an item set to its frequency. Confidence is the so-called interestingness measure for ARs and indicates the estimated conditional probability of 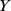 given 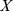. Traditionally, AR mining consists of pruning all items with the support below a user-defined minimum support threshold and subsequent pruning of rules whose confidence is below a user-defined minimum confidence threshold [6]. The proper choice of the support and confidence thresholds can become a challenging problem for the user because it severely affects the size of the found rule set.

Generally, traditional AR mining algorithms such as, e.g., Apriori [7] with the standard support-confidence approach, generate a huge amount of associations, which are largely redundant. This is an essential drawback for biological applications [8], [9]. One of the reasons is that support filtering eliminates low support rules, classifying them as uninteresting. This is due to the fact that in market basket analysis, it may not be reasonable to pay attention to the products that are rarely bought together. When investigating biological data, however, we are – in contrast to the market basket analysis – interested in finding rare associations that affect only a small set of proteins rather than frequent ones which often correspond to well-known facts. Inside this reduced set, the association can be strong and informative enough for meriting our attention [10] even if it does not meet the minimum support criterion. A concept of rare cases [11] is therefore especially useful for those applications where support filtering would eliminate infrequent but interesting relations. Rare cases correspond to a meaningful but relatively small subset of the data. Consequently, a rare AR is defined as a rule connecting either frequent and rare items or only rare items [12].

To improve the mining of rare ARs, special properties of interestingness measures with respect to rare items should be taken into account. Recently, several criteria were defined for selecting an appropriate interestingness measure for rare ARs [12]. The most important of them is the null-transaction invariance, which means that a measure is not affected by the number of null-transactions, i.e. transactions that contain none of the items of interest. Moreover, for several well-known measures [13] the lack of suitability for rare AR mining have been shown there. It was stated that only Cosine, All-Confidence and Jaccard measures should be considered for mining rare ARs because they are null-transaction invariant. However, it can be shown that 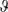, for example, is not a null-transaction invariant but converges to the certain value with an increasing number of transactions (see Supplemental Material S1 for the proof). For this reason, 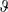 was also included in the set of analyzed measures.

Unfortunately, none of the discussed interestingness measures, which are well-suited for mining rare ARs, can treat hierarchically organized data in an appropriate manner. In such a case, the redundancy of ARs is caused to a large extent by the hierarchical structure itself because the rules in higher hierarchy levels subsume the rules in deeper levels. Thus, the hierarchy can be naturally used for removing redundant rules. The approach is partly implemented in the hierarchical pruning method of [6], which we later refer to as Generalized Rule Pruning (GRP). Srikant et al. extended the standard support-confidence framework for hierarchical rules also called Generalized Association Rules (GARs) (i.e. rules that can span different hierarchy levels). Srikant et al. also proposed pruning more specialized rules deeper in the hierarchy unless they differ significantly from their ancestor rules as measured by calculating expected values for support and confidence. This enables significant reduction of the found rule set as compared with standard AR mining in the presence of a hierarchy. However, GRP has been initially developed for market basket analysis and therefore it cannot address the specificity of bioinformatics problems where the associations of interest are sparse and rare. In this manner, the root nodes of a hierarchy, for example, are always considered by GRP as interesting. This is an essential drawback in respect to GO. Additionally, it uses the confidence measure, which is inappropriate for mining rare rules. Our goal was therefore to overcome these limitations by developing special interesting measures, which can rank rare rules higher if they are interesting in terms of hierarchy. The proposed class of *Interestingness by Difference* achieves this goal. It is based on calculating expected values in a manner similar to GRP, but is general enough to be applied also to those measures that are well-suited for mining rare ARs as well, and not only to support or confidence. Thus, it can be useful in mining rare and hierarchical rules. We claim that the proposed approach can be especially helpful for connecting biological ontologies, because here the aim is to find interesting rules, which could not otherwise be derived simply from the hierarchy.

This study was in part presented in our conference paper [14] in which we published some preliminary results as well as a short analysis of two datasets, also used here: DBpedia-Yago and GPCR-GO (the latter with different preprocessing than here). In this paper, however, we focus on the problem of mining rare rules and examine the results in more detail. Moreover, we apply our method to an additional dataset [15], compare our approach with two alternative AR mining methods GRP and GRL, and provide a more comprehensive performance evaluation.

### Related work

Despite its popularity in the data mining community, association analysis has not yet become an established data analysis tool in bioinformatics. And yet, rising interest in AR mining has been noted over the last decade, for example in the analysis of micro-array data. Several studies have been recently conducted to find groups of co-expressed genes by means of association analysis as an alternative to widely used clustering methods [3], [8], [16]–[20]. In this context, ARs can describe relations between expression levels of genes and certain cellular conditions – which genes are overexpressed or underexpressed in diseased cells as compared to healthy ones, for example. The most promising studies integrate a priori biological knowledge (e.g. metabolic pathways or GO categories) into the rule mining process with the aim of utilizing as much available information as possible [3], [8].

Association analysis was applied to the problem of finding errors in electronically assigned functional annotations in large sequence annotation databases [21], [22]. Another interesting application is the search for predictive combinations of genes in the genotype-phenotype relationships [23], [24]. One of the most recent applications of association analysis is presented in [9] where classical AR mining is combined with a novel approach to identify indirect associations and hidden biological regularities by using semantic-preserving vocabulary and association networks.

Two tasks that come close to ours are presented in [25] and [15]. The former applied the standard Apriori algorithm to connect 238 GO terms (i.e. only a small part of the data) of three GO branches: Molecular Function (GO-MF), Cellular Component (GO-CC) and Biological Process (GO-BP) by cross-ontology rules. The same task was previously addressed in [26] by three different approaches: the first one based on similarity in the vector space, the second one based on the statistical analysis of co-occurrences of GO terms, while the third also dealt with AR mining in the standard setting. To the best of our knowledge, the most recent work in the area of cross-ontology rule mining was reported in [27], [28]. Both approaches were developed explicitly for mining cross-ontology multi-level association rules between three GO branches. The first approach uses the bottom-up generalization of rules level-by-level and a Monte Carlo simulation for its termination. It applies the Apriori algorithm at each iteration. The second one generalizes GO terms to all their ancestors and requires only a single pass through the Apriori algorithm. A standard Apriori implementation with support, confidence and Chi-square thresholds was used in both cases. Additionally, several pruning criteria, e.g. for cross-ontology or ancestor rules, were employed for removing closely related, irrelevant or known rules. More general rules were also pruned unless their confidence difference as compared to a child rule was greater than 10%. There was neither reason for the choice of the certain value nor recommendations for the parameter setting given.

In the study of Faria et al. [15] the GO Relationship Learning (GRL) algorithm was presented recently in order to find inconsistent GO annotations. GO-MF annotations were mined for pairwise ARs connecting single GO terms. GRL differs from the standard AR mining algorithm by a so-called agreement parameter, which is utilized along with the confidence and is essentially the same but takes only unique combinations of GO-terms (so-called MF classes) into account instead of all proteins. It also applies an additional rule filtering (referred here to as Specific Structure Pruning (SSP)) to the found rule set. It prunes all rules where the antecedent and the consequent are connected by four or less edges, all rules whose items have more than 10 descendants and all ancestor rules (i.e. it prunes a rule if an ancestor of the antecedent or the consequent of another rule appears in it). The drawbacks of this method are the somewhat arbitrary SSP parameter setting adapted for the special dataset and an additional user-defined threshold. The choice of parameters can become a great challenge for the user if GRL is to be applied to another dataset, even if this is at some future point in time, as GO changes its structure constantly. This method is especially interesting for comparison with the proposed approach because it also uses pairwise ARs, hierarchical pruning and a large dataset. For this reason it was chosen along with GRP for the experimental comparison.

To the best of the authors' knowledge, all of the studies considered so far are based on the standard AR mining within the support-confidence framework. None of them search for rare associations, nor do they exploit alternative interestingness measures. We fill this gap by using the proposed approach.

## Materials and Methods

In this study we propose a novel approach to rare AR mining, which takes the specificity of bioinformatics problems into account, in order to cope with the biological ontologies. The proposed approach is able to solve the following task. We are given a set of objects 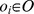, which are classified into a set of classes given by multiple ontologies 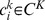: 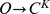. If an object belongs to a certain category, it should also belong to all of its ancestors. The task is to derive pairwise associations between the categories of different ontologies, i.e. to find out how two categories 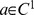 and 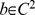, 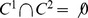, 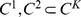 are related. A transaction in this case is represented by an object that belongs to 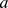 and 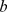 simultaneously. This task is a frequently found setup in multi-label classification, when an object can belong to several class taxonomies [29] at the same time.

In GO, the most specific categories are relatively infrequent in comparison with their ancestors, however the associations between the most specific categories could be of significance as the categories of high levels tend to produce trivial rules. Another supporting argument is that the assignment of GO categories is generally a well-considered process as opposed to the stochastic process of filling transactions in the market basket analysis. As we intend to find the most interesting connections between ontologies and not merely the most frequent and perhaps most obvious, we investigated all those associations with at least one occurrence. For a similar reason, multiple-level AR mining [30] cannot be used to solve our task, because it assumes the manual selection of the support threshold at each hierarchy level separately. This would be difficult for the user, on the one hand, and could hide rare but interesting rules, on the other.

The disadvantage of the methods discussed above is the use of the confidence measure. It was heavily criticized as being unable to extract truly interesting rules [31] because of its major drawback - the inability to detect statistical independence or negative dependence between items [32]. Additionally, the confidence measure was shown to fail the important criteria for mining rare ARs [12]. As rare associations are more difficult to detect and use to generalize from, due to less data, appropriate methods are needed, e.g., special interestingness measures. For these reasons, we developed a special class of hierarchical interestingness measures *Interestingness by Difference* shown at the bottom of Table 1. In contrast to GRP, where only expected values (as calculated with respect to a parent rule in the hierarchy) of support and confidence are used, hierarchical measures can be based on the expected value of any interestingness measure with the range of [0,1]. In GRP, the expectations are defined as a fraction of the *Sup* (*Cnf*) of the parent rule proportional to the child-parent *Sup* ratio. Although in this study we adopted this definition of the expectations, *Interestingness by Difference* metrics can in principle conform to alternative definitions. Srikant et al. proposed for GRP to retain only those rules, whose actual values of *Sup* (or *Cnf*) significantly exceed their corresponding expectations. To achieve this, a user defined threshold was introduced, bounding GRP to its appropriate setting.

**Table 1:** Measure List.

Number	Measure name	Abbreviation	Formula	Range	Ref.
1.	Support	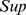	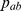	[0,1]	[7]
2.	Confidence	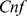	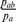	[0,1]	[7]
3.	Cosine	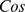	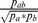	[0,1]	[12]
4.	All-Confidence	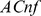	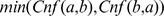	[0,1]	[12]
5.	Kulczynski	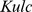	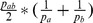	[0,1]	[38]
6.	Lift	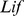	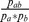	[0, 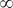 )	[43]
7.	Bayes Factor	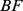	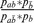	[0, 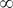 )	[13]
8.	CenteredConfidence	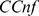	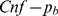	[−1,1)	[13]
9.	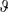 -coefficient	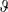	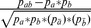	[0,1]	[44]
10.	Jaccard	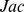	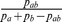	[0,1]	[44]
11.	JacDif	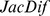	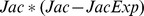	(−1,1]	our
12.	CosDif	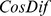	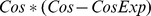	(−1,1]	our

The list of the used interestingness measures and abbreviations. Term 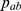 refers to the fraction of transactions from the whole transaction set where 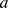 and 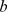 co-occurred, analogically for 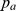 and 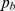.

In our method, no threshold is needed when comparing the real value 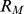 of an interestingness measure 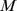 for a rule 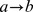 with the expected value 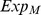. *Interestingness by Difference* is then defined as:
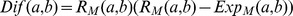



If expected values become greater than the real ones, *Dif* converts to negative. This happens, for example, when a sibling or even the parent of a node has a stronger relation to the consequent of the rule. The *Dif* approach can be applied to different interestingness measures, but here we will use only *JacDif* and *CosDif*. The main motivation for this decision is that we are interested in finding rare association rules, on the one hand, and the terms which are equally important to each other, on the other hand. Both aspects are well covered by these both measures.

We calculate the expectations substituting 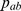 with the support expectation 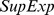, as implied by Srikant et al. in their calculation of confidence expectation. Taking into account a generalization of a rule on both antecedent and consequent sides, 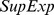 can be defined as follows:
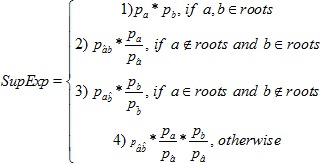
(1)


where 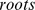 is the set of the root nodes of both ontologies and 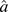 () is the parent of 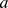 (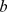) within the respective hierarchy. Note that the handling of root nodes is different to by Srikant et al. as we use the independence assumption here. In contrast, GRP always defines rules involving the root nodes as interesting. If there are multiple parents (e.g. the hierarchy is a Directed Acyclic Graph (DAG)), only the smallest expectation value over all parents is kept.

If only the left side hierarchy is taken into account for simplicity, Equation 1 reduces to the cases one and two. So, for example, the expectation of *Jac* will be, for non-root nodes:
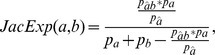



In this case, the expectation expresses the *Jac* value of the child's rule 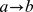 which is expected on the basis of the *Jac* value of the parent rule 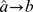 and the distribution of instances between the parent and the child. The generalization on both sides and its comparison with the generalization only on the antecedent side we leave for future work.

**Figure 1:**
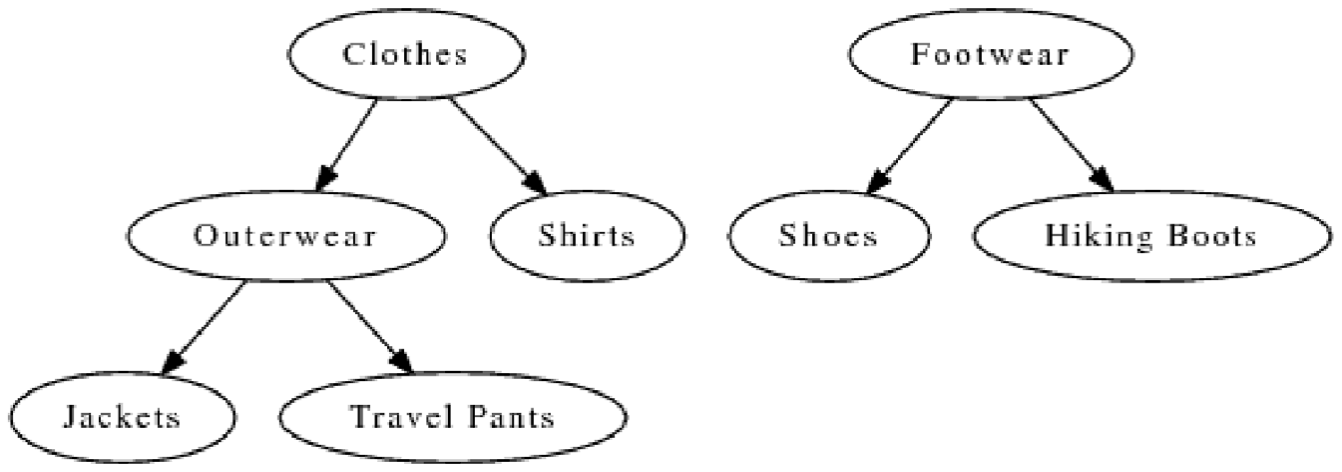
Example hierarchy.

To better explain the role of expectations, we can involve the example presented by Tables 2 and 3, and Figure 1. As one can see from Rule 3, the *SupExp* value is higher than the actual *Sup* value and 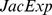 is greater than 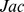. This causes the 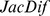 value to be negative. Rule 4 has a higher 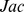 value than Rule 3 but its 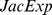 is much lower. Thus, its 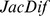 achieves a higher value. Since the expectation is calculated based on the original assumption that the distribution of transactions between children and their parent holds with respect to the items of a rule, a higher actual value points to a stronger correlation than expected. Such rules are unexpected from the point of view of the hierarchy and should be ranked higher than the rules like Rule 3.

**Table 2:** Hierarchical Measures Example.

Nr.	Rule	Support	Item	Support
1	Clothes 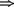 Hiking Boots	30	Clothes	150
2	Outerwear 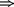 Hiking Boots	20	Outerwear	100
3	Jackets 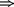 Hiking Boots	15	Jackets	90
4	Travel Pants 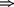 Hiking Boots	10	Travel Pants	15
			Hiking Boots	30

**Table 3:** Hierarchical Measures 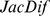 and 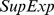 Example.

Nr.	Rule	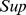	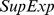	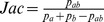	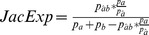	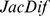
3	Jackets 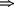 Hiking Boots	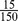	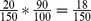	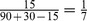	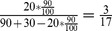	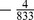 = −0.005
4	Travel Pants 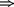 Hiking Boots	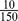	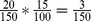	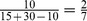	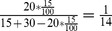	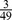 = 0.06

### Data

We first used the dataset DBpedia-Yago from [33] with two similar ontologies and a set of manually created rules connecting them (the so-called ground truth set). It is based on the entries of DBpedia, which were also tagged by Yago's labels. DBpedia and Yago are both based on Wikipedia data, but Yago is extended with other data sources like WordNet and GeoNames and uses the data differently to construct its ontology. Both ontologies are DAGs. As a ground truth set was used a partial gold standard mapping between DBpedia and Yago with 151 links. (http://www.netestate.de/De/Loesungen/DBpedia-YAGO-Ontology-Matching, there was 169 rules but only 151 appeared in the data.) Unfortunately, it covers only a small part of possible true relations between the ontologies. Our dataset had 271 and 97,680 labels of DBpedia and Yago, respectively. The total number of instances (Wikipedia websites) was 159,889. Each instance had the mean number of labels of 4.4 for DBpedia and of 9.3 for Yago.

The second dataset – G Protein-Coupled Receptor - Gene Ontology (GPCR-GO) – contained proteins from the GPCRDB database (http://www.gpcr.org/7tm/) with a tree-like hierarchy. Each of the proteins was downloaded from the UniProtKB (http://www.uniprot.org/uniprot/) on October 4th 2013 to check which GO terms were assigned to it (excluding annotated electronically, which are denoted as IEA). The IEA terms were not included in the analysis since we wanted to extract confirmed GO terms. We separated the GO branches: Biological Process (GO-BP), Cellular Component (GO-CC), and Molecular Function (GO-MF) by creating different subsets called GPCR-GO-BP, GPCR-GO-CC, and GPCR-GO-MF for each of them. Here, only the results of the latter subset are reported. Note that they deviate from the previously published results [14] because of different preprocessing. Only the nodes of the GO hierarchy, which were actually assigned to the proteins, and their respective ancestor nodes were analyzed. The maximal depth of GO-MF hierarchy was ten levels, of GPCR it was only 6. In this dataset, there were 2442 proteins and 407 GO-MF as well as 729 GPCR terms involved.

The third dataset GO-GO was constructed similarly to that of [15] in order to compare obtained ARs. However, there were some small deviations, as we could obtain not exactly the same support values but only very close ones for the rules listed. We could reproduce the extraction of rules and their ranking to a large extent. In this dataset, the GO-MF annotations were gathered from the UniProtKB for every protein of the database in October 2010. Additionally, we constructed a recent dataset in May 2013 in order to analyze the differences in annotations occurred during this period, for example, corrected errors. We also gathered the respective GO structure for the corresponding time frame. Each annotation was expanded by its ancestors from the GO ontology. In October 2010, about 6 million proteins had a GO-MF annotation corresponding to 8892 different GO-MF terms. In May 2013, there were already more than 19 million proteins with 9568 different GO-MF terms. In this dataset, the proteins with the IEA assignments were also allowed to be able to compare to and reproduce the results of [15].

To convert each one of the DAG hierarchies of DBpedia-Yago into a tree, for every node with multiple parents we created a new node for each parent, copying the descendants and assuring that each node had only one parent. In the GO-MF-GPCR and GO-GO dataset the created tree would be too large, therefore we used the DAG structure there.

All data were downloaded from the online sources, parsed and preprocessed with customized python scripts and imported into Matlab where our own implementation of the Apriori algorithm with all discussed interesting measures and pruning methods was applied to the data.

## Results and Discussion

The proposed method was experimentally compared with standard AR mining by several popular interestingness measures shown in Table 1 as well as with the methods GRP and GRL (assuming that obtained rules are ranked by *Cnf*). The measures sensitive to rare associations were the focus of the comparison. The ground truth set of the dataset DBpedia-Yago was first exploited for the performance comparison of the interestingness measures. Such an approach is often used to validate results [34], [35] because it is typically not known how many and what type of relations should be discovered. The number of discovered true associations was employed as an indicator of the measure's quality. Next, the proposed hierarchical measures along with GRP and GRL as well as with four measures well-suited for mining rare ARs were applied to two bioinformatics datasets.

### Finding the True Connections: DBpedia-Yago

In the first experiment, the performance of the proposed approach in comparison with different interestingness measures as well as with GRP and GRL was assessed by the 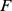-1 measure which is the harmonic mean of precision and recall. Table 4 presents the number of extracted rules, the number of the true rules among the extracted rules, and also the corresponding F-1 value. In the upper part of the table, only 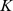 top ranked rules are retained, and 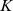 is chosen to be equal to the number of the true rules. The bottom area of the table shows the results of the “best possible” setting. In this case, the best possible size of a found rule set with respect to the obtained 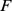-1 value is searched for iteratively. To this end, all rules are first sorted by their measure values in descending order, whereupon 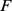-1 is calculated and the last rule (with the lowest value) is removed from the set. The next iteration starts with recalculating the 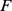-1 value. Finally, the rule set with the highest 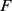-1 score is selected.

**Table 4:** DBpedia-Yago performance results.

Method	*Cnf*	*Jac*	*Cos*	*ACnf*	*Kulc*	*Lift*	*BF*	*CCnf*	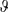	GRP	GRL	*JacDif*	*CosDif*
Best 151													
T-Rules	7	74	73	74	72	5	7	29	73	7	54	73	68
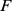 -1	4.64	**49.01**	**48.34**	**49.04**	47.68	3.31	4.63	19.21	**48.34**	4.64	35.76	**48.34**	45.03
Best possible													
Rules	609	225	201	208	187	265	447	384	199	591	107	195	221
T-Rules	105	96	89	92	85	10	33	91	89	105	54	90	94
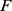 -1	27.63	**51.06**	50.57	**51.25**	50.30	4.81	11.04	34.02	50.85	28.30	41.86	**52.02**	50.54

DBpedia-Yago: The number of found rules and the number of true rules among them (T-rules), for the first 151 and for the best possible rule set. 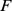-1 is in %. Three best values are shown in bold.

*ACnf*, *Jac*, *Cos*, 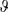 and *JacDif* had the best three 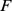-1 values in the first setting. All these interestingness measures are well-suited for mining rare ARs. Among the others *Kulc* was the best. In the “best possible” setting, *JacDif*, *ACnf*, and *Jac* had even better results. *CosDif* had slightly lower performance values, but they were higher than those of the conventional measures *Cnf*, *CCnf*, and *BF*. *Lift* had the worst 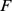-1 values in both experiments.

GRP could not improve the result of *Cnf* for the first 151 rules (since they have basically the same rule ranking), but it had a slightly better result in the “best possible” setting. Overall it had relatively low 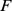-1 values. For the GRL pruning method, we used its standard setting with the minimum absolute support (the number of instances is referred in this paper as absolute support, also called as support count) of 10 co-occurrences, minimum confidence (*min Cnf*) of 0.8 and agreement (*min Agr*) of 0.8. Additionally, the ancestor rules were removed, leaving only 107 rules. Notice that SSP cannot be entirely applied when connecting two different ontologies, only the limitation to nodes with less or equal 10 descendants. The result of GRL was much better than that produced by *Cnf* or even GRP, but it was still worse than the results of other measures.

**Figure 2:**
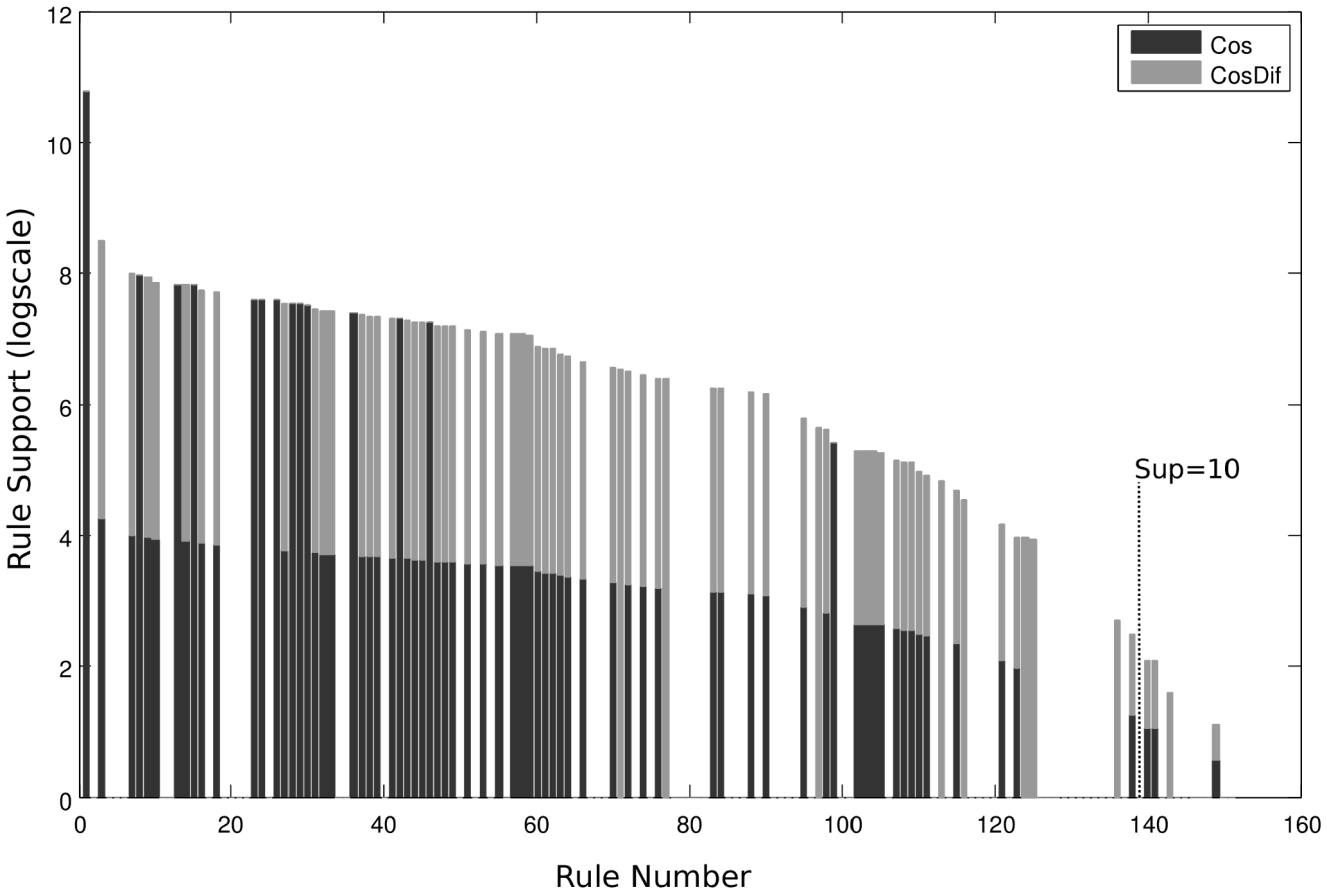
Sorted true rules by absolute support. The color indicates if it was in the first 151 rules of each metric.

To analyze rare rule mining more precisely, we focused on the support distribution of the true rules found in the first experiment. Figure 2 shows the true rules found by *CosDif* and *Cos*, ranked by their support values. The main advantage of *CosDif* over *Cos* is that although *CosDif* found five true rules fewer; it found four more rules than *Cos* with an absolute support value below 55 – a strong evidence for rare rules. Notice that GRL with its default setting would be unable to find the rules with the absolute support value below 10. In fact, the lowest absolute support among the true rules it found was equal to 53. We tried to set the absolute minimum support in GRL to the unity what led to the discovery of four true rules (out of 13) with the absolute support values below 10. In this case the total number of the found true rules increased to 56. Table 5 shows median absolute support values for all methods in the first experiment. The median was chosen over the mean because there was a single true rule with very large absolute support. In contrast, the relation between the corresponding median values of *JacDif* and *CosDif* was much more balanced. It is also important to note that the absence of minimum support filtering was critical for some measures like *Cnf* and *Lift*. They ranked high many uninteresting low-support rules that were absent in the true rule set, mostly describing a hierarchical relationship like DB:BadmintonPlayer
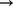
YG:player110439851 (rank 4, with 1 instance). Such hierarchical relationships can be described by 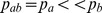.

**Table 5:** Median absolute support of “best 151 rules” the DBpedia-Yago dataset.

Method	*Cnf*	*Jac*	*Cos*	*ACnf*	*Kulc*	*Lift*	*BF*	*CCnf*	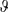	*JacDif*	*CosDif*	GRP	GRL
found true rules	53	1202	1204	1193	1202	3	53	1150	1200	942	696.5	53	1030
not found true rules	635.5	378	359	435	378	635.5	635.5	579	406.5	496	551	635.5	480
all found rules	4	1222	1222	1082	1204	1	4	3	1204	779	547	4	591

Median absolute support of the rules in the setting “best 151 rules”.

Table 6 shows the number of common rules among the best 500 rules as found by the pairwise comparison of studied methods. The best 500 rules were taken since lower values had too few samples, meaning that some of the methods did not intersect at all. Furthermore, a higher number of rules did not cause any remarkable change in the relations described. This is valid for all datasets. One can see from the Table 6 that the maximum intersection between the rule sets of *Cnf* and GRP. A large number of rules was also shared by the pairs from *Jac*, *Cos*, *ACnf*, *Kulc*, and 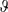. *JacDif* and *CosDif* had 462 common rules, which is even more than they shared with their conventional counterparts. The fewest number of intersections with other methods had GRL because of its SSP resulting in pruning many rules.

**Table 6:** The number of intersections for DBpedia-Yago.

	*Jac*	*Cos*	*ACnf*	*Kulc*	*Lift*	*BF*	*CCnf*	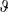	*JacDif*	*CosDif*	GRP	GRL
*Cnf*	211	211	209	207	43	218	451	210	203	188	490	107
*Jac*		479	472	441	82	144	244	470	459	427	215	71
*Cos*			451	461	83	145	244	488	453	430	215	71
*ACnf*				416	83	145	242	445	444	406	213	71
*Kulc*					97	156	236	465	431	428	211	69
*Lift*						317	48	94	97	103	42	2
*BF*							224	156	158	161	217	30
*CCnf*								244	230	212	456	00
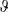									458	437	214	71
*JacDif*										462	206	70
*CosDif*											191	67
GRP												103

The number of intersections among the best 500 rules extracted by different methods from the DBpedia-Yago dataset.

The more detailed comparison of the true rules found by *JacDif* and *Jac* among the best 151 can explain the difference in their rule sets. Table 7 shows the different rules, their support and their ranking by both metrics. *JacDif* extracted more rare association rules since the rules found by it and not by *Jac* had lower support. As can be seen, although *JacDif* found one true rule fewer than *Jac*, there were 9 different true rules in total. The rules not discovered by *JacDif* typically had a high expectation and were therefore penalized by the metric. For example, Rule 5 was expected from the hierarchy due to the parent DB:EducationalInstitution
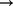
YG:educationalinstitution108276342. Indeed, some of the true rules that can be derived from the hierarchy e. g. DB:Game 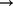 YG:game100456199 (*Jac* rank 177, *JacDif* rank 506) with the parent DB:Activity 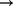 YG:contest107456188 were ranked by *JacDif* lower as uninteresting. In principle, such rules can be ignored by an expert when reviewing the extracted rules because they do not deviate from their parents. An important characteristic of the true rules found by *Jac* was their high support, which was in complete contrast to the rules found by *JacDif* and those not found by *Jac*. Thus, *JacDif* was able to discover more specific rules as compared with *Jac*. An example was the rule DB:SpaceStation
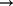
YG:Spacestations ranked 142 by *JacDif*. This choice seems to be quite reasonable because the parent of DB:SpaceStation was DB:MeanOfTransportation and the rule DB:MeanOfTransportation 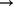 YG:Spacestations would be too general (rank 45,429).

**Table 7:** Difference in the true rules sets of *Jac* and *JacDif* for the DBpedia-Yago dataset.

Nr.	Sup(a,b)	Sup(a)	Sup(b)	DBpedia	Yago	rank Jac	rank JacDif
The true rules found by *Jac* but not *JacDif*							
1	1847	2521	1847	Planet	planet109394007	134	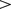 400,000
2	1974	1997	1984	RadioStation	radiostation104044119	27	569
3	1864	1884	1879	River	river109411430	30	359
4	1396	1552	1657	Saint	saint110546850	126	152
5	2553	3065	3045	School	school108276720	145	188
The true rules found by *JacDif* but not *Jac*							
1	93	149	93	Archaea	Archaeagenera	176	151
2	5	8	5	Continent	Continents	173	149
3	15	23	15	SpaceStation	Spacestations	163	142
4	51	64	60	Valley	valley109468604	153	117

The difference in the found true rules of *Jac* and *JacDif* among the best 151. Support refers in this table to the absolute support (number of instances: sup*N).

### GPCR-GO

In this experiment, there is no ground truth rule set to be discovered, as the method of connecting the ontologies is not obvious. The number of rules extracted by each method is depicted in Table 8. In our analysis we focused mostly on the top 200 rules. Only the associations between GPCR and GO-MF were examined in the experiment. The MF branch was chosen in order to narrow the analysis and because the molecular function is closer to GPCR hierarchy, which is based on the pharmacological classification of GPCRs [36]. Nevertheless, connecting both ontologies was a challenging task because of their different points of view. For example, GPCR and GO have several entries related to hormones, but whereas GPCR always connect the term to a protein (group), like “hormone protein” or “gonadotropin-releasing hormone”, the branch GO-MF has more abstract terms and links them to the function, not to a certain protein type or group, like: “regulation of hormone levels”, “juvenile hormone secretion”, “hormone transport”, etc. Moreover, the complete structure of GO-MF (with 10 levels) is more dense connected and deeper than that of GPCR. Another relevant difficulty with this dataset is that although the GPCR-families are well annotated – despite the absence of secondary functions of the sequences – there are several missing, inconsistent GO-term assignments or the assigned terms are too broad. This inconsistency is caused primarily by varying GO knowledge of experts and by the fact that not all proteins were tested for each possible GO-term.

**Table 8:** Median absolute support and the number of intersections for GPCR-GO-MF dataset.

	Nr. of Rules	Median Sup.	*Cos*	*ACnf*	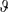	*JacDif*	*CosDif*	GRP	GRL
*Jac*	8781	5	459	444	450	414	371	11	31
*Cos*	8781	5		420	487	412	375	13	31
*ACnf*	8781	6			409	393	342	8	31
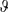	8781	4				417	379	13	33
*JacDif*	7879	5					443	12	21
*CosDif*	7077	4						12	17
GRP	5419	3							49
GRL	511	15							

Median absolute support and the number of intersections among the best 500 rules extracted by different methods from the GPCR-GO-MF dataset (for GRL only 32 rules).

All discussed issues led to a considerable number of trivial rules ranked high by all methods. One example of an evident rule was GPCR: “Serotonin”
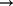
GO-MF:“serotonin receptor activity”, which was not extracted by GRL because of its ancestor removal (GRL extracted the more specific rule GPCR:“Serotonin 1”
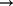
GO-MF:“serotonin receptor activity”). *JacDif* ranked this rule higher than the other methods. GRP ranked it low (2037th). The rankings for this and other discussed rules are depicted in Table 9.

**Table 9:** Rule Rankings for GPCR-GO-MF dataset.

Rule	*Jac*	*Cos*	*ACnf*	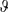	*JacDif*	*CosDif*	GRP	GRL
GPCR:Serotonin 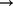 GO-MF:“serotonin receptor activity”	42	43	43	43	36	45	2037	
GPCR:“Chemokine receptor-like” 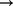 GO-MF:“steroid hormone receptor activity”	129	130	142	114	76	60	2421	
GPCR:“Anaphylatoxin” 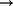 GO-MF:“anaphylatoxin receptor activity”	43	42	42	42	23	19	2035	
GPCR:P2RY1 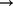 GO-MF:“nucleotide binding”	989	1159	970	1128	1329	1780	2553	
GPCR:“Trace amine” 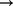 GO-MF:“G-protein coupled receptor activity”	3055	2276	3175	2714	7466	6671		485

This rule did not cover all proteins that were tagged as GPCR:“Serotonin”. There were three proteins not connected to the GO-MF:“serotonin receptor activity”: 5HT6R_HUMAN, Q9W3V5_DROME and Q9VEG1_DROME. We would like to suggest that this was probably a missing annotation in GO-MF. It is clear for 5HT6R_HUMAN since this protein is also known as Serotonin receptor 6 and obtained the corresponding annotation in April 2013 from IEA:Compara method. This example shows that it would be useful to examine such strong rules in the context of why they do not cover all proteins with the antecedent annotation since this often points to an annotation inconsistency [15], [21]. Thus, our approach could assist GO curators in the inconsistency detection and the assignment of missing GO terms to the GPCR proteins.

Our approach could predict several correct GO annotations. For example, the rule GPCR:“Chemokine receptor-like”
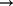
GO-MF:“steroid hormone receptor activity” was ranked much higher by *CosDif* (rank 60) and *JacDif* (rank 76) than by the others. The pruning methods ranked it very low: GRP ranked this rule 1230th and it was not found at all by GRL. There were two proteins that supported this rule: GPER_RAT and B3G515_DANRE. However, in total there were three items assigned to GPCR:“Chemokine receptor-like”. The one missing protein was GPER_HUMAN (also known as Q99527). It was not annotated by the GO term “steroid hormone receptor activity” at the time the data were gathered nor did it have any other manually curated term. The only GO-MF term assigned to it at this time was “G-protein coupled receptor activity” and it was an IEA term. In the version of October 16th 2013 it obtained the term GO-MF:“estrogen receptor activity”, a child of “steroid hormone receptor activity”, by inference from direct assay [37].

Three measures well-suited for mining rare ARs: *Jac*, *Cos*, and *ACnf* extracted similar sets of rules as can be seen on the example of 500 top ranked rules in Table 8. This similarity appears because in the context of rare items, *ACnf* and *Jac* formulas tend to produce similar results, since if 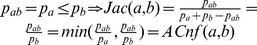 where 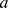 and 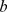 can be exchanged. This is given in this dataset because of the relatively low number of proteins involved (with manually curated GO annotations). *Cos* and 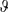 also had similar sets since 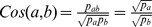 has similar ordering as 
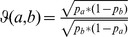
 under the given conditions. The rule set of 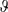 differed a little more from *Jac* and *ACnf* mainly because there is a difference in weighting due to the square root in the formulas of *Cos* and 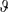. Also the sets of *JacDif* and *CosDif* had more similarity with each other than with any of the other methods.

The rule sets extracted by GRP and GRL did not have any substantial overlap with the other sets. While GRP extracted mostly rules where the absolute support was equal to 1 (because of the lack of the minimum support threshold), the other methods had rules with a higher support as can be seen from the slightly higher median absolute support (Table 8). GRL could only extract 511 rules that matched its requirements mostly because of minimum support filtering (only 960 rules in this dataset had absolute support that was over 10) and because of the agreement threshold. This can be explained by the inappropriate use of the parameter values chosen initially for the GO-GO dataset. Thus, the need for a trial-and-error approach to choosing the parameters is a clear disadvantage of GRL. Another limitation is its inability to find rare rules. The median absolute support of GRL was the highest, indicating that more general rules were extracted. Indeed, many high ranked rules were connected to the GO term “receptor activity”, which is trivial in this experiment and provides no useful information. Another group of extracted rules involved the GO terms with the suffix “signal transducer activity”, which was also high placed in the hierarchy. These rules were extracted even though the ancestor removal step of GRL was supposed to prune them, as the lower level rules did not achieve the minimum agreement or support and were therefore discarded even before this step could take place.

In contrast, *JacDif* generally ranked rules higher if they could be seen as more surprising from the hierarchy. This is illustrated by the rule GPCR:“Anaphylatoxin”
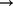
GO-MF:“anaphylatoxin receptor activity” ranked 23th as compared with the rule GPCR:Serotonin
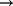
GO-MF:“serotonin receptor activity”, which was ranked only 36th. The former rule was ranked higher because its expectation was equal to 0.03 whereas the actual *Jac* value was 0.94. The serotonin rule, in turn, had the same *Jac* value, but its expectation was much higher (0.14).

In the top 20 rules extracted by *JacDif* about half of the rules were also in the top 20 from *Jac*, *Cos*, *ACnf* and 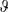. From these about half were predicted from the hierarchy and the other half was high ranked by *JacDif*. *CosDif* found four other rules in comparison to *JacDif*, but they were high ranked by *JacDif*. So, both measures had similar rule sets. GRP had only two rules in common with *JacDif* in its 20 top rules. This is rather a coincidence since there were 1994 rules with *Cnf* equal unity in the rule set extracted by GRP, thus all these were ranked as most interesting. GRL did not have any common rules with *JacDif*. GRL had also a high number of highest ranked rules, 412 with *Cnf* equal one, thus every comparison using the first 20 top ranked rules would be not appropriate. Most of these 412 rules found by GRL obeyed the hierarchical relationship (more than 350 rules sufficed the relation 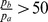) which is typical for a child-parent relation of ontologies. Such associations will usually not lead to discovering any new knowledge since they present obvious facts. This confirms the merit of using an alternative to *Cnf* in order to extract rare and interesting rules.

### GO-GO

In this experiment we first investigated the AR mining methods' ability to discover inconsistent rules, i.e. rules that correspond directly to inconsistent annotations. The second step involved comparing the quality of the top ranked rules extracted by our method with the rules from [15]. Further, the annotation data were gathered in May 2013 and compared with the data from October 2010.

### Inconsistent rules

**Figure 3:**
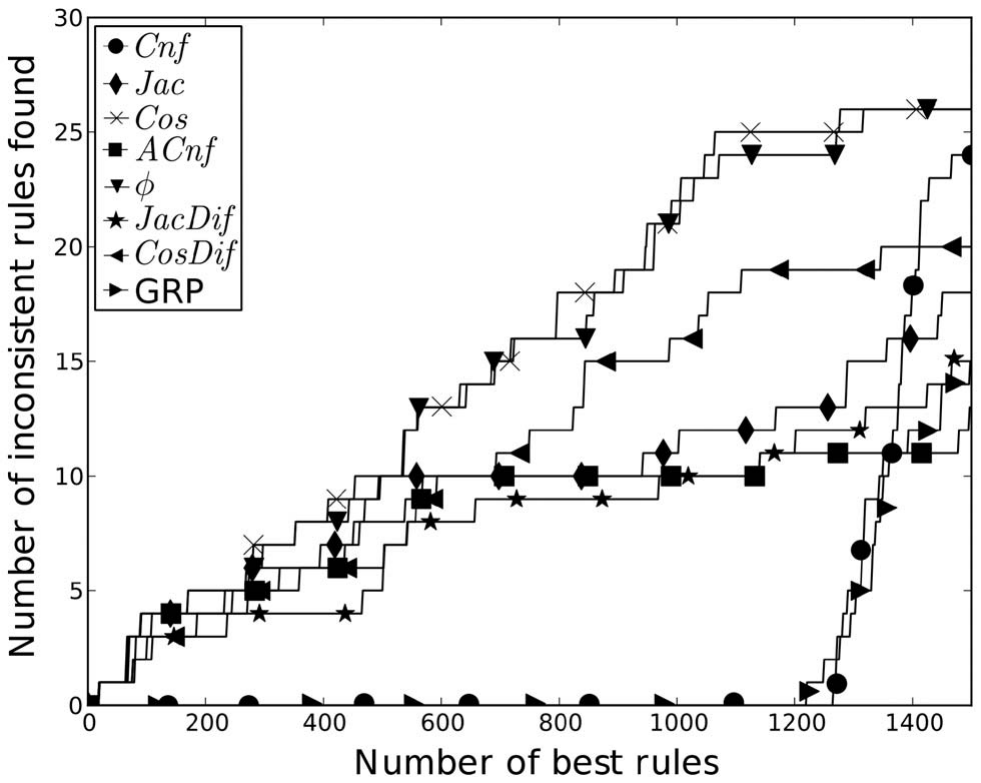
Number of inconsistent rules. The number of inconsistent rules found in the best x rules extracted by the given metric. The best x rules were gathered from 20,000 and then ancestors rules were removed.

First of all we were interested in examining how sensitive to inconsistent annotations is our method as compared with GRL, GRP, and traditional interestingness measures. Specially because the goal of GRL was to find inconsistent GO annotations. However, it should be noted that the aim of our method was different and we therefore did not expect very good results. To this end, the table of manually specified inconsistent “MF classes” (Supplemental Material S2) provided by Faria et al. was analyzed and those containing only two MF terms were converted into the AR form. We naturally excluded 13 MF classes of the table denoted by the statement “no inconsistencies found”. As a result, a set of 63 inconsistent rules with pairwise relations was produced. Next we applied different AR mining methods to search for rules in the dataset. We compared the rules extracted by *Jac*, *Cos*, *ACnf*, 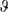, *JacDif*, *CosDif*, GRL and GRP. In Fig. 3 the number of inconsistent rules found among 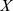 top ranked rules is depicted. To select the best rules, the best 10,000 were taken for each method. Each of these rules was supposed to have occurred at least once; only one direction was allowed (from two directions a rule can have, when exchanging antecedent and consequent, only the higher ranked one was chosen) and SSP was applied. One can see that until 500 all metrics had almost the same number of discovered rules (around 8). Afterwards *Cos* and 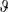 discovered more inconsistent rules than the rest and achieved 26 rules by 1300 total rules. The *Dif* based measures did not find as many inconsistent rules mainly because many of them (18) were expected by the hierarchy.

The GRL method with the following parameters: *min Cnf* = 0.8, *min Agr* = 0.7, *min Sup* = 10/N, and SSP, pruned all but 513 rules from 408,662. There were 25 inconsistent rules among them. This result is better than the result obtained by the compared interestingness measures and well as by GRP. This is largely due to the complex preprocessing, which compensated for the limitations of *Cnf*. Indeed, there were about 1,300 rules with the unity *Cnf* value and no inconsistent rules among them. So, *Cnf* needed many more rules than the other methods to discover its first inconsistent rule. GRP took almost the same number of rules to discover its first inconsistent rule as *Cnf*. The poor discriminancy of *Cnf* confirms the argument that it is not an appropriate metric for solving this task. However, GRL can significantly improve it as our experiment showed.

### Top ranked rules

The analysis of the Table S3 from [15] with the ARs found by GRL in this dataset demonstrates another problem with *Cnf*. All of the 100 top-ranked rules reported there (extracted by *Cnf* and sorted by the decreasing *Sup*) are highly asymmetrical: only eleven of them show a *Cnf* in the opposite direction of more than 0.1. This means that an antecedent is often connected to a very frequent term – “ATP binding”, for example. This behavior is similar to that of GRL on the previous dataset and may be useful for the search for inconsistent annotations when a missing term is supposed. However, in general, there is no clear evidence whether such asymmetrical rules may be reliably considered as confident [38]. Furthermore, in such relations the antecedent is connected only with a part of the consequent that is the feature of a specific-to-general relationship of an ontology as discussed above. Although GRL could not detect any rare associations in the first 100 rules, one should note that they were sorted by *Sup* and are therefore not directly comparable with the rules of other methods, which are sorted by their interestingness measures.

To overcome this problem, we analyzed the best 500 rules of GRL sorted by *Cnf* in the descending order (Table 10). One can see that GRL found the rules with a much higher support as compared with the other methods. Its median absolute support was 146 (compare with the value of 5 of most of the other methods). In its 500 rules, only 101 had the absolute support below 20, whereas for *JacDif* there were 342 such rules. The small number of intersections between the sets of found rules also shows that GRL detected different rules as compared with the other methods. One of the reasons for this is the agreement parameter, which is designed to identify item sets that often relate to each other. One of its properties is that it favors more general terms higher in the hierarchy as they occur frequently. Many small inconsistencies of the protein annotation can have a considerable impact on the number of assigned unique GO-term sets to which a GO-term belongs. This results in the decrease of the agreement down in the hierarchy. The most common rules were extracted by *Cos* and 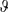: their rule sets were almost identical. The rule set of *Jac* was also very similar to them, while the rule sets of *JacDif* and *CosDif* were very similar to each other, even more similar than to each of their counterparts.

**Table 10:** Median absolute support and the number of intersections for GO-MF-GO-MF.

Metric	Nr. of rules	Median Sup.	*Cos*	*ACnf*	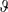	*JacDif*	*CosDif*	GRP	GRL
*Jac*	5445	5	433	436	433	227	206	47	11
*Cos*	5485	6		379	498	203	230	53	16
*ACnf*	5518	5			379	197	176	39	10
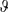	5495	5.5				203	231	53	16
*JacDif*	5678	5					431	33	14
*CosDif*	5717	5						38	14
GRP	5688	2							67
GRL	513	146							

Median absolute support and the number of intersections between the best 500 rules extracted by different methods from the GO-MF-GO-MF dataset. Nr. of rules refers to the total number of rules after preprocessing.

To analyze the quality of the rules obtained by *JacDif* more precisely, we focused on the first 20 of them (Table 11, Supplemental Material S1). We were able to prove that all 20 rules were true. One can see that our approach did not extract asymmetrical rules, however it was able to detect rare associations: all 20 rules have a relatively symmetrical nature and eleven rules show an absolute support below 10. It seems that they connect very specific terms and thus are not used frequently. For instance, the GO terms of Rule 3 are consecutive reactions and therefore the combination of both activities is reasonable. Furthermore, the GO terms of Rule 4 “transport arabinose” and “fucose” are both monosaccharides. Even though the transporter specificity is normally quite high (one or a few), it is reasonable to assume that more proteins can transport arabinose and fucose interchangeably than annotated so far. Especially since the reference publication [39] for the co-occurrence of both reports that L-galactose and D-arabinose can also be transported. Furthermore, according to a BLAST (blastp) search [40], the gene for L-fucose transporter is present in many species of bacteria.

**Table 11:** 20 best rules extracted by *JacDif* the GO-MF dataset.

Nr.	*Sup*	*Cnf*	*JD*	GO name	GO name	*Sup*13	10-13	13-10
1.	5	1	1.00	GO:0008954 peptidoglycan synthetase activity	GO:0016807 cysteine-type carboxypeptidase activity	0	5	0
2.	1	1	1.00	GO:0034437 glycoprotein transporter activity	GO:0034041 sterol-transporting ATPase activity	19	0	18
3.	2	1	1.00	GO:0010490 UDP-4-keto-rhamnose-4-keto-reductase activity	GO:0010489 UDP-4-keto-6-deoxy-glucose-3,5-epimerase activity	4	0	2
4.	1	1	1.00	GO:0015518 arabinose:hydrogen symporter activity	GO:0015150 fucose transmembrane transporter activity	1	0	0
5.	1	1	1.00	GO:0070905 serine binding	GO:0010855 adenylate cyclase inhibitor activity	20	0	19
6.	1	1	1.00	GO:0050241 pyrroline-2-carboxylate reductase activity	GO:0050132 N-methylalanine dehydrogenase activity	2	0	1
7.	1	1	1.00	GO:0017045 adrenocorticotropin-releasing hormone activity	GO:0051431 corticotropin-releasing hormone receptor 2 binding	3	0	2
8.	1	1	1.00	GO:0017045 adrenocorticotropin-releasing hormone activity	GO:0051430 corticotropin-releasing hormone receptor 1 binding	3	0	2
9.	9	1	1.00	GO:0047376 all-trans-retinyl-palmitate hydrolase activity	GO:0050251 retinol isomerase activity	0	9	0
10.	2	1	1.00	GO:0035473 lipase binding	GO:0035478 chylomicron binding	13	0	11
11.	2	1	1.00	GO:0080048 GDP-D-glucose phosphorylase activity	GO:0010475 galactose-1-phosphate guanylyltransferase (GDP) activity	8	0	6
12.	1130	1	0.99	GO:0043752 adenosylcobinamide kinase activity	GO:0008820 cobinamide phosphate guanylyltransferase activity	1518	812	1,202
13.	3590	1	0.97	GO:0004743 pyruvate kinase activity	GO:0030955 potassium ion binding	11,356	188	7,975
14.	2407	1	0.87	GO:0004643 phosphoribosylaminoimidazolecarboxamide formyltransferase activity	GO:0003937 IMP cyclohydrolase activity	7759	83	5,438
15.	1756	0.99	0.86	GO:0019134 glucosamine-1-phosphate N-acetyltransferase activity	GO:0003977 UDP-N-acetylglucosamine diphosphorylase activity	6889	77	5,211
16.	2424	0.97	0.85	GO:0004486 methenyltetrahydrofolate dehydrogenase activity	GO:0004477 methylenetetrahydrofolate cyclohydrolase activity	4	2,418	0
17.	329	0.93	0.85	GO:0051861 glycolipid binding	GO:0017089 glycolipid transporter activity	935	14	624
18.	1862	0.95	0.84	GO:0004633 phosphopantothenoylcysteine decarboxylase activity	GO:0004632 phosphopantothenate–cysteine ligase activity	6009	86	4,234
19.	1619	0.84	0.67	GO:0008066 glutamate receptor activity	GO:0005234 extracellular-glutamate-gated ion channel activity	3967	69	2,435
20.	2074	0.86	0.65	GO:0008531 riboflavin kinase activity	GO:0003919 FMN adenylyltransferase activity	7022	78	5,028

20 best rules extracted by *JacDif* (*JD*) with Faria's filtering method without filtering by *min Sup*, *min Cnf* and *min Agr*.

Rule 5 was explained in [41]: mGluR4, -7, and -8 are negatively coupled to adenylate cyclase when expressed in hamster ovary cells. These mGluRs are also selectively activated by l-serine-O-phosphate [42]. These studies form the basis for assigning the items of Rule 5 to GRM7_HUMAN. The glutamate receptor, metabotropic 7 marker, also appears in 19 other proteins found in the dataset of 2013. This confirms the high evidence of the rule. Rules 7 and 8 are trivial, GO:0017045 describes a hormone and GO:0051430 or GO:0051431 the corresponding receptors (adrenocorticotropin is also known as corticotropin). Rule 9 describes again the consecutive reactions.

The rules in the lower part of the table have much higher support, since they are mostly IEA based on InterPro rules. For a detailed analysis see Supplemental Material S3.

We also analyzed the annotation data from May 2013 in order to examine whether the rules extracted from the data of 2010 could be confirmed by future annotations. Indeed, for most of the rules the support increased considerably (Table 11). The differences in the table should not sum up since the numbers may contain doubles. We used the protein name and not the accession number, this is relevant in only a few cases. Most of the first eleven rules have more co-occurrences in 2013 than in 2010 what confirms their biological merit. The support decreased only for Rules 1, 9 and 16. The antecedent of Rule 1 was declared obsolete and the new replacement (GO:0071972) also has five co-occurrences with the consequent of that rule in the data of 2013. Rule 9 was based on the gene RPE65 of several species. The antecedent and the consequent of the rule were replaced by their siblings (GO:0042574
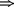
GO:0052884, 5 co-occurrences). Rule 16 had GO:0004486 in the antecedent since this GO term was the parent of GO:0004488 in 2010 and was assigned to many proteins supporting the rule. In 2013, the former GO term became just the sibling of the latter, thus the concerned proteins covered by GO:0004488 do not support the rule anymore.

The consequent of Rule 12 was removed from IPR003203, which explains the drop of many proteins from this rule (since the IEA rule does not apply anymore as before). On the other hand, the consequent was assigned to many proteins with this antecedent based on the EC 2.7.7.62 (EC2GO with the base protein P0AE76), i.e. from another IEA source. The base protein (Q05599) of IPR003203 also has this consequent (stand of 19th of August 2013), and is cross-referenced in EC 2.7.7.62. This explains the total increase of proteins covered by that rule.

Rules 13–15 and 17–20 probably follow the same pattern: some proteins were removed (deleted from the database) and many new ones obey the rules. So, one can see that only a few rules discovered from the data of 2010 do not hold anymore mainly because of ontology changes. Such ontology changes are common and provide an additional reason why inconsistency analysis should be conducted in large datasets.

Comparing the sets of the first 20 best rules of the other methods to those of *JacDif*, the sets extracted by the metrics were relatively similar. *ACnf* differed by only three rules, *Jac*, *Cos* and 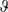 by four. As before *JacDif* favors rules that are unexpected in terms of the hierarchy, therefore this is where the difference tends to lie between the sets of the metrics. The sets extracted by the methods GRP and GRL were totally different in comparison with what was extracted by *JacDif*. Again, the best rules extracted by the methods GRL and GRP had a specific to general character, but there were many rules with *Cnf* that equaled the unity value (for GRP 1214 and for GRL 177). The metrics tried to find rules where 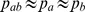 therefore they were better at discriminating, i.e. there were not many rules with the highest score.

## Conclusion

In this paper we examined relating multiple biological ontologies by association analysis. Associations found between classes of different ontologies can be used to support existing knowledge or to extract new knowledge with the aim of better understanding biological mechanisms. We focused on mining rare associations which are very important for the biological understanding of the data as they describe relations that are not obvious and difficult to find. To this end, a new class of interestingness measures – *Interestingness by Difference* – was especially developed for hierarchically organized rules. These measures favor rules with a large difference between their actual and expected interestingness values. The expectation is calculated taking into account the parent rule. So, the rules that are unexpected from the hierarchy can be ranked higher even if they are rare.

The proposed approach was applied to three real-world datasets, two of which are from the bioinformatics domain. The approach was first compared with several conventional interestingness measures, with measures well-suited for mining rare rules as well as with two hierarchical pruning methods GRP and GRL on the dataset with the underlying ground truth rule set. The results showed that the proposed approach was able to extract more true rules than the other methods.

The analysis of rules obtained for the GPCR-GO and GO-GO datasets showed that redundant hierarchical rules were pruned and interesting rare rules were ranked higher. Conventional AR mining methods would miss them in the huge amount of rules that can be extracted. Our analysis also revealed that the rules higher ranked by the proposed method are meaningful associations connecting certain proteins which make sense biologically. Some other rules are very promising, but will need further investigation. Even a prediction of a future annotation's assignment was possible by the proposed method. Such GPCRDB family prediction leads to the assumption that this additional information source would enrich the InterPro predictions. Additionally, discovered ARs enable a database curator to find inconsistencies in the actual data and methods.

## Supporting Information

Supplemental Material S1
**Concise proof that if the number of transactions increases to infinity and the number of transactions including a and including b are fixed the** 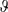 **value for that rule will converge to** 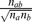 **. This term is null-invariant.**
(PDF)

Supplemental Material S2 **Spreadsheet with rules from GO-GO section.** They also include the evidence discussed in the text and in S3.
(ODS)

Supplemental Material S3 **Detailed discussion of rules extracted in the GO-GO section.**
(PDF)
