# Correction: Mining Rare Associations between Biological Ontologies

**DOI:** 10.1371/journal.pone.0103663

**Published:** 2014-07-25

**Authors:** The PLOS ONE Staff

There is an error in the first equation. Please see the corrected Equation 1 here.



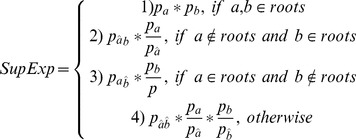
(1)

