# Supplementary material for: Mining Rare Associations between Biological Ontologies
Source: PLoS One. 2014 Jan 3;9(1):e84475. doi: 10.1371/journal.pone.0084475 (PMC3880308; doi:10.1371/journal.pone.0084475)
Supplement: Supplemental Material S1 — Concise proof that if the number of transactions increases to infinity and the number of transactions including a and including b are fixed the value for that rule will converge to . This term is null-invariant. (PDF) [file pone.0084475.s001.pdf]

Supplemental Material to "Mining Rare Associations between Biological Ontologies": Proof ϕ null-invariance

Fernando Benites[1,*], Svenja Simon[1], Elena Sapozhnikova[1]
1 Department of Computer and Information Science, University of Konstanz, Konstanz, Germany
∗ E-mail: Fernando.Benites@Uni-Konstanz.de

Proof ϕ converges to null-invariant

$$\lim_{n\to\infty}\phi = \lim_{n\to\infty}\frac{\frac{n_{ab}}{n}-\frac{n_a}{n}\frac{n_b}{n}}{\sqrt{\frac{n_a}{n}\frac{n_b}{n}\frac{n-n_a}{n}\frac{n-n_b}{n}}} = \frac{n_{ab}}{\sqrt{n_a n_b}}$$

n is the number of transactions, n_{ab} the number of transactions where item a and b appear together, n_a (n_b) the number of transactions where a (b) appears. $n_a, n_b \neq 0$.
