# Supplementary material for: Mining Rare Associations between Biological Ontologies
Source: PLoS One. 2014 Jan 3;9(1):e84475. doi: 10.1371/journal.pone.0084475 (PMC3880308; doi:10.1371/journal.pone.0084475)
Supplement: Supplemental Material S3 — Detailed discussion of rules extracted in the GO-GO section. (PDF) [file pone.0084475.s003.pdf]

Supplemental Material to “Mining Rare Associations between Biological Ontologies”: Rule evidences

Fernando Benites[1,*], Svenja Simon[1], Elena Sapozhnikova[1]
1 Department of Computer and Information Science, University of Konstanz, Konstanz, Germany
∗ E-mail: Fernando.Benites@Uni-Konstanz.de

Analysis of JacDif Rules Evidences for GO-GO

The underlying biological common aspect we could identify was that four protein families (Rule 14, 15, 18 and 20) are bifunctional and each catalyze two GO terms. Rule 14: The AICARFT/IMPCHase bienzyme (IPR002695[1]) catalyzes the last two steps in de novo purine biosynthesis. Rule 15: The bifunctional UDP-N-acetylglucosamine pyrophosphorylase/glucosamine-1-phosphate N-acetyltransferase (IPR005882) is a bifunctional enzyme that catalyzes the last two sequential reactions in the de novo biosynthetic pathway for UDP-GlcNAc. Rule 18: The coenzyme A biosynthesis bifunctional protein CoaBC (IPR005252) catalyzes the second and third steps in the biosynthesis of coenzyme A (CoA). Rule 20: Bacterial Riboflavin kinase (IPR002606) is another bifunctional protein. Interestingly, eukaryotes usually have two separate enzymes. Rule 13: Pyruvate kinase (IPR001697) is an example of a protein family where ions are needed for function. Both magnesium and potassium ions are needed for catalysis of the final step in glycolysis. Rules 12 (IPR003203) and 16 again combine consecutive reactions. By contrast, Rule 17 (IPR014830) is trivial connecting glycolipid transporter activity and glycolipid binding. Similarly, Rule 19 (IPR019594 has the child of the antecedent and the consequent) combines glutamate receptor activity and extracellularglutamate-gated ion channel activity. GO even states in the comments of GO:0005234 “Consider also annotating to the molecular function term: 'glutamate receptor activity; GO:0008066'.”

The explanations we found for Rule 1, 2, 6, 10 and 11 are more restricted. Rule 1 (the base protein for the rule (BPR): YBIS_ECOLI[2]), Rule 2 (BPR: ABCG1_HUMAN; [1], [2]), and Rule 11 (BPR: GGAP1_ARATH and GGAP2_ARATH; [3], [4], [5]) are based on well studied single proteins whose functions have been propagated through pattern matching and IEA to other proteins. The latter two rules have antecedents and consequents that come from at least two different studies depending on the base protein. Rule 6 was predicted by two different EC[3] templates. Rule 10 (BPR: GPIHBP1) was examined in [6]). Nevertheless, for four of these rules (all but Rule 6) there are a few reviewed co-annotations of both terms and the rules seem to be plausible, since most of them have a reference protein where all aspects have been verified.

References

1. Tansley GH, Burgess BL, Bryan MT, Su Y, Hirsch-Reinshagen V, et al. (2007) The cholesterol transporter abcg1 modulates the subcellular distribution and proteolytic processing of -amyloid precursor protein. Journal of Lipid Research 48: 1022-1034.

2. Engel T, Kannenberg F, Fobker M, Nofer JR, Bode G, et al. (2007) Expression of {ATP} binding cassette-transporter {ABCG1} prevents cell death by transporting cytotoxic 7-hydroxycholesterol. {FEBS} Letters 581: 1673 - 1680.

[1] IPRXXXXXX stand for a unique InterPro (http://www.ebi.ac.uk/interpro/) accession number
[2] http://www.uniprot.org/uniprot/P0AAX8.txt?version=41
[3] Enzyme Commission http://enzyme.expasy.org/

3. Linster CL, Adler LN, Webb K, Christensen KC, Brenner C, et al. (2008) A second gdp-l-galactose phosphorylase in arabidopsis en route to vitamin c: Covalent intermediate and substrate requirements for the conserved reaction. Journal of Biological Chemistry 283: 18483-18492.

4. Linster CL, Gomez TA, Christensen KC, Adler LN, Young BD, et al. (2007) Arabidopsis vtc2 encodes a gdp-l-galactose phosphorylase, the last unknown enzyme in the smirnoff-wheeler pathway to ascorbic acid in plants. Journal of Biological Chemistry 282: 18879-18885.

5. Dowdle J, Ishikawa T, Gatzek S, Rolinski S, Smirnoff N (2007) Two genes in arabidopsis thaliana encoding gdp-l-galactose phosphorylase are required for ascorbate biosynthesis and seedling viability. The Plant Journal 52: 673–689.

6. Gin P, Beigneux AP, Davies B, Young MF, Ryan RO, et al. (2007) Normal binding of lipoprotein lipase, chylomicrons, and apo-AV to GPIHBP1 containing a G56R amino acid substitution. Biochim Biophys Acta 1771: 1464–1468.
